# Heat exposure and maternal stress: evidence from the GRAPHS pregnancy cohort in Ghana

**DOI:** 10.1088/2752-5309/ae90dc

**Published:** 2026-08-25

**Authors:** Lewis White, Kenneth A Ae-Ngibise, Cascade Tuholske, Darby Jack, Mohammed N Mujtaba, Robbie M Parks, Michelle Li, Steven Chillrud, Andrew Zimmer, Seyram Kaali, Oscar Agyei, Dana C Jack, Eqi Luo, Alison Lee, Kwaku Poku Asante

**Affiliations:** 1 Department of Environmental Health Sciences, Mailman School of Public Health, Columbia University, New York, NY, United States of America; 2 Kintampo Health Research Centre, Research and Development Division, Ghana Health Service, Kintampo, Ghana; 3 Department of Medicine, Division of Pulmonary, Critical Care and Sleep Medicine, Icahn School of Medicine at Mount Sinai, New York, NY, United States of America; 4 Department of Earth Sciences, Montana State University, Bozeman, MT, United States of America; 5 Geospatial Core Facility, Montana State University, Bozeman, MT, United States of America; 6 Lamont-Doherty Earth Observatory, Columbia University, New York, NY, United States of America; 7 Western Washington University, Bellingham, WA, United States of America

**Keywords:** heat exposure, maternal stress, pregnancy, distributed lag non-linear model, Ghana

## Abstract

Heat exposure has been linked to psychosocial stress, an established antecedent of perinatal depression; however, evidence on heat-related stress during pregnancy in sub-Saharan Africa remains limited. We analyzed psychosocial stress scores and covariate data from the Ghana Randomized Air Pollution and Health Study, linking daily maximum and minimum shaded wet bulb globe temperature (WBGT) metrics to participants’ stress scores derived from the Crisis in Family Systems-Revised Life Events Questionnaire. We evaluated associations using ordinal logistic regression of pregnancy-average and trimester-average exposures and distributed lag non-linear models (DLNMs) to assess time-varying associations across gestation. Higher average maximum WBGT exposure across pregnancy was associated with increased odds of higher psychosocial stress; each 1 °C increase in maximum WBGT was associated with 64% higher odds of belonging to a higher stress category (OR = 1.64; 95% CI = 1.17–2.31; *p* = 0.0040). In trimester-average models, higher first-trimester maximum WBGT was also associated with higher stress (OR = 1.44; 95% CI = 1.15–1.81; *p* = 0.0014). DLNMs suggested that relatively cooler daily maximum WBGT values (25th percentile) were associated with decreased odds of stress in early pregnancy, whereas extreme daily maximum WBGT values (99th percentile) showed a pattern consistent with increased odds of stress in mid-to-late gestation. These findings highlight gestational windows in which heat exposure may influence stress, emphasizing the need for further research into underlying mechanisms and effective interventions to protect maternal mental health in heat-vulnerable settings.

## 1. Introduction

A substantial body of evidence links extreme heat exposure to a range of adverse health outcomes, from direct heat-related illnesses such as heat exhaustion and heatstroke to the exacerbation of chronic cardiovascular and respiratory diseases [1, 2]. Growing evidence also links heat exposure to adverse mental health outcomes [3]. However, links between heat exposure and mental health outcomes, especially during susceptible windows such as pregnancy, remain incompletely characterized, particularly in sub-Saharan Africa. Understanding these relationships is increasingly important as anthropogenic climate change intensifies the frequency and duration of extreme heat events, acting as a threat multiplier for existing health and social vulnerabilities [4].

Higher ambient temperatures have been associated with adverse mental health outcomes, including increased suicide risk [5]. Proposed mechanisms include thermoregulatory stress, sleep disruption, and reduced coping capacity, which are also relevant to more common conditions such as depression and anxiety [2, 6].

Pregnant women are more vulnerable to heat exposure compared with the general population [7]. Mental health burdens are already substantial in many African settings. In Ghana, for example, an Accra-based study found over 25% of sampled women reported depressive symptoms [8]. Physiological changes during pregnancy, such as alterations in hormone balance and thermoregulation, can heighten sensitivity to heat exposure [7]. In households with more traditional gender roles, as is common in Ghana, women’s domestic and caregiving responsibilities may persist or be only partially redistributed during pregnancy [9]. Together, these physiological and social factors may increase vulnerability to temperature-related stress. Consistent with this heightened vulnerability in the perinatal period, a study of new mothers in the Bawku municipality of Ghana found that 50% of the sample experienced postpartum depression [10].

Evidence from sub-Saharan Africa illustrates how extreme heat can affect women’s health and well-being, particularly during pregnancy and the perinatal period. In a 2024 ethnography study in Burkina Faso, women described how extreme heat compromised their physical, psychological, social, and economic wellbeing [11]. Heat limited their ability to rest and care for infants, intensified irritability and conflict within households, and reduced capacity to work or earn income. Such findings emphasize how environmental stress can simultaneously strain women’s physical health, emotional well-being, and social relationships. These stressors not only affect maternal well-being but also influence child outcomes. A 2020 systematic review reported that heat wave exposure during pregnancy was associated with 16% higher odds of preterm birth, with higher temperatures also associated with increased odds of stillbirth [12]. In addition, maternal stress has been documented to be associated with preterm birth, low birth weight, and adverse neurodevelopment for the child [13].

Despite growing evidence that heat exposure affects mental health, research at the intersection of heat and maternal mental health is sparse, particularly when focusing on sub-Saharan Africa [3, 14]. Moreover, much of the existing research focuses on pregnancy as a single exposure window, rather than evaluating critical periods where higher heat exposures may be more connected to stress [12, 13]. As a result, the timing and cumulative effects of temperature-related stress during pregnancy remain poorly understood.

To address these gaps, we examined whether heat exposure over pregnancy and the perinatal period is associated with psychosocial stress among women in Ghana. We leveraged the Ghana Randomized Air Pollution and Health Study (GRAPHS) to assign daily wet bulb globe temperature (WBGT), which integrates temperature and humidity to capture physiological heat stress in humid climates such as Ghana, across pregnancy. We utilized the Crisis in Family Systems-Revised (CRISYS-R) questionnaire to assess psychosocial stress across multiple domains [15]. We then employed distributed lag non-linear models (DLNMs) to examine time-varying associations between shaded daily maximum and minimum WBGT and maternal stress. Finally, we conducted sensitivity analyses using other established heat stress metrics [16], including heat index (HI), 2 m dry air temperature (T2m) and wet-bulb temperature (Tw).

## 2. Study design and methods

### 2.1. Study participants

Psychosocial stress data were collected from mothers enrolled in the GRAPHS. GRAPHS, described in Jack *et al* (2015), is a cluster-randomized trial designed to assess the health impacts of clean cooking interventions [17]. From August 2013 to March 2016 1414 pregnant women in the Kintampo North and Kintampo South districts were recruited. Eligible women were in their first or second trimester, carrying a live singleton fetus, identified as the primary household cook, and were non-smokers. Since enrollment, GRAPHS has collected health, environmental, and demographic data for participating mothers and their children. Follow-up data collection is ongoing to support analyses across a range of health outcomes. The current analyses include a subset of GRAPHS mothers who additionally completed stress assessment questionnaires from 2014–2015.

### 2.2. Stress measurement

We measured stress in a convenience sample subset of pregnant women from the GRAPHS cohort using the CRISYS-R Life Events Questionnaire [15], which assesses exposure to significant life events across 12 domains during the previous six months. For each item, participants indicated whether the event occurred, and if so, whether their emotional response was positive, negative, or neutral. Events are grouped into 12 domains (e.g. financial, relationship, medical, etc). A negative domain score (NDS) was calculated as the number of domains in which a participant reported at least one negatively perceived event. These scores were then classified into low (NDS = 0–2), moderate (NDS = 3–5), and high (NDS > 5) stress, matching previous analyses using this data [18]. This ordinal stress variable served as the primary outcome for the analysis. For a supplementary analysis, the number of negatively perceived financial events—the highest reported domain—was additionally categorized into ordinal groups (0, 1–2, and ⩾3 events).

### 2.3. Exposure metric

Hourly ERA5 reanalysis data [19] (≈25 km resolution) was used to derive daily minimum, maximum, and mean WBGT for each participant’s home community. First, we estimated hourly relative humidity (RH) with ERA5 T2m and dewpoint temperature [20] in order to calculate HI with T2m and RH following the U.S. National Weather Service procedure [21]. WBGT was then estimated from HI values using the quadratic transformation described in Bernard & Iheanacho (2015), providing WBGT estimates expressed in °C under shaded conditions (i.e. no direct solar radiation) and assuming a fixed wind speed (0.5 m s^−1^) [22]. WBGT was selected as our primary heat-stress metric because it integrates air temperature and humidity into a single index that more closely reflects physiological heat stress on the human body [23].

For each participant, daily maximum and minimum WBGT values were calculated and averaged over dates corresponding to the preconception period, each trimester, and the entire pregnancy window. Daily minimum WBGT, which largely reflects nighttime temperature, was used to examine the impact of nighttime heat exposure. In addition, overnight cooling was characterized as the difference between daily maximum and minimum WBGT (ΔWBGT), reflecting the extent of overnight temperature relief. HI, T2m, and Tw from ERA5 data were evaluated in sensitivity analyses, as described below.

The centroid of each community was used to determine the corresponding ERA5 grid cell, so all participants within the same community had the same exposure at matching dates. Ten unique grid cells comprised all 41 communities represented in the data. However, because exposure was aligned to each participant’s conception date, and conception dates ranged from November 2013 to February 2015, participants within the same grid cell were generally assigned different daily WBGT sequences over their respective gestational windows. Model-based standard errors are reported as primary inference, as the analytical unit is the individual participant; a cluster bootstrap sensitivity analysis (resampling at grid-cell level, 1000 replications) is reported in the supplementary materials.

### 2.4. Covariates

To account for potential confounding, models adjusted for several maternal characteristics and contextual factors: age (continuous), education level (categorical), marital status (categorical), body mass index (BMI; continuous), socioeconomic status (SES; ordinal), parity (continuous), and season of delivery (wet [April–October] vs dry [November–March]). SES was derived from an asset-based index used across GRAPHS studies [24]. Due to sparse cell counts in some categories, marital status was modeled as a binary indicator of partnered versus not partnered, and education level was collapsed into three categories (none, primary, junior high school or further education). These covariates were selected because they may influence both maternal stress and environmental heat exposure. To assess potential collinearity between heat exposure and seasonal adjustment, generalized variance inflation factors (GVIF) were calculated for the WBGT term under each seasonal specification. Sensitivity analyses refitting all primary models under alternative seasonal/calendar-time specifications (calendar month of conception; sin/cos harmonic terms for conception day-of-year; no seasonal adjustment) were conducted to assess robustness of findings to seasonal adjustment choice. A directed acyclic graph depicting our assumed causal structure is provided in supplementary figure S1.

### 2.5. Statistical analysis

Primary analyses comprised two complementary components, alongside supporting descriptive analyses. First, exposure-window analyses examined five pre-specified periods—preconception, first trimester, second trimester, third trimester, and entire pregnancy—using daily maximum WBGT and ordinal logistic regression. To address multiplicity within this discrete family of exposure-window models, Benjamini–Hochberg FDR-adjusted *p*-values were calculated as a sensitivity check alongside the primary unadjusted estimates, with Bonferroni-adjusted *p*-values additionally reported as a more conservative bound. Second, DLNM analyses were used to characterize the timing and shape of associations between daily maximum and minimum WBGT and stress across gestation. Because DLNMs estimate continuous exposure–lag–response surfaces rather than a set of independent tests at each lag or percentile, interpretation focused on the overall timing, direction, and consistency of the estimated associations rather than isolated pointwise statistical significance; simultaneous Scheffé confidence bands were computed alongside pointwise 95% confidence intervals. Supporting analyses, including domain-specific stress outcomes, overnight cooling models, alternative heat metrics, percentile contrasts, and DLNM specification checks, were interpreted descriptively as sensitivity or model-characterization analyses.

Prior to modeling, we examined distributions of stress outcomes, heat exposure, and covariates using descriptive statistics and graphical diagnostics. Stress categories were well populated and heat exposures showed plausible ranges without extreme outliers. The proportional odds assumption was assessed using the Brant test and verified empirically by recoding CRISYS-R scores as binary at both outcome thresholds and refitting models as logistic regression. To explore temporal patterns, we first fit ordinal logistic regression models to examine associations between trimester average heat exposure and maternal stress. Separate models were fit using the mean of daily maximum WBGT values during the 30 d preconception period, each trimester, and across the entire pregnancy. We additionally fit a model that considered the preconception period and each trimester-specific average heat exposure simultaneously.

To examine time-varying associations, we next applied a DLNM framework [25, 26]. DLNMs allow for assessment of both the magnitude and timing of heat–stress associations across gestation, and potential non-linear associations. Daily maximum and daily minimum WBGT were examined as primary exposures in DLNMs. As a sensitivity analysis, we fit a joint DLNM including cross-basis terms for daily maximum WBGT and overnight cooling (ΔWBGT) to assess robustness of lag patterns. We also fit a DLNM focusing specifically on the financial stress domain, as this was the most common contributor to NDS scores. To facilitate interpretation, cumulative associations were summarized over 7 d lag windows, representing short-term heat exposure patterns, rather than across the entire exposure period.

For all DLNMs, the cross-basis was specified using natural cubic splines for both the exposure (4 degrees of freedom) and lag (3 degrees of freedom) dimensions. DLNM spline specifications were selected through a two-stage process. First, 97 specifications (B-spline and natural spline combinations across varying degrees of freedom) were screened using BIC; natural splines, which also enforce linearity at the boundaries of the exposure distribution and avoid implausible extrapolation at extreme WBGT values, consistently achieved lower BIC and were selected. Second, the four scientifically appropriate candidate specifications (var df ⩾ 3, lag df ⩾ 3) were compared visually using lag-response surfaces, with the ns(4,3) specification selected as the primary model on the basis of stability across the full WBGT range and biologically plausible behavior. Full details of the selection process, including BIC values for the candidate specifications and visual comparisons of candidate surfaces, are provided in the supplementary materials. Lag-response surfaces for all four candidate specifications are shown in supplementary figures S2 and S3, and cumulative ORs at highlighted gestational windows were consistent in direction across all four candidate specifications (supplementary table S1), indicating that the primary finding is not an artifact of specification choice. The median of the pooled daily maximum WBGT values across all participant-days (27.4 °C) was used as the DLNM reference value, representing typical within-population exposure; this differs slightly from the participant-level summary (27.2 °C, the median of individual participants’ mean daily maximum WBGT). Seven-day cumulative windows were used to summarize short-term heat exposure patterns; sensitivity to both the reference value and window length is reported in supplementary tables S2 and S3.

We defined the exposure period as 30 d prior to conception through 210 d after conception (two weeks into the third trimester), when the questionnaire was typically given. This window maximized temporal coverage while minimizing sample loss due to incomplete exposure histories. DLNM estimation required a balanced dataset in which each participant contributed the same number of days of exposure; therefore, participants who completed the survey before accumulating sufficient follow-up time (e.g. early in the second trimester or near the start of the third trimester) were excluded, as exposure after the date of stress assessment cannot be meaningfully assigned.

All models (regressions and DLNMs) adjust for the covariates described above. As a sensitivity analysis, all primary models were repeated using alternative heat exposure metrics (HI, T2m, and Tw). These analyses followed the same model structure, lag period, and covariate adjustment as the primary WBGT models.

### 2.6. Ethics approval and informed consent

The GRAPHS cohort was conducted with written informed consent from all participants, in accordance with the principles of the Declaration of Helsinki and local statutory requirements. Study procedures were approved by the Kintampo Health Research Centre Institutional Ethics Committee (KHRCIEC/2017–31), Ghana Health Service (GHS-ERC: 032/03/23), and the Institutional Review Boards at Columbia University (IRB-AAAR4373) and the Icahn School of Medicine at Mount Sinai (STUDY-17-01265).

### 2.7. Software

ERA5 data was extracted using Python v 3.11. All statistical analyses were conducted in R version 4.3.1. We used the MASS package for ordinal logistic regressions and conducted the distributed lag non-linear models via the DLNM package.

### 2.8. Role of the funding source

The funders had no role in study design, data collection, data analysis, data interpretation, or writing of the report. The corresponding author had full access to all the data and had final responsibility for the decision to submit for publication.

## 3. Results

Of the 354 women who completed the stress survey, 41 were excluded because they completed the survey before reaching 210 gestational days and did not contribute the full exposure window needed for the DLNM or for trimester-specific WBGT estimates; one additional participant was excluded for missing BMI, yielding a final analytic sample of 312. Of these, 302 (96.8%) were surveyed at least two weeks into the third trimester and before delivery, while 10 (3.2%) were surveyed postpartum, with a median of 1.1 weeks after delivery (IQR: 0.7–2.8 weeks; range 0.1–4.1 weeks), and were included, as the CRISYS-R captures life events over the prior six months.

Participants had a median age of 28 years (IQR: 23–34) and a median BMI of 22.9 kg m^−2^ (IQR: 21.2–24.7). The distribution of parity—the number of times a woman has given birth to a fetus that has reached at least 24 weeks, regardless of the pregnancy outcome—was skewed right, with a median of 3 and an IQR of 1–5. SES, defined via an asset index, was roughly evenly represented across the sample. Most participants were either married (57%) or living with their partner (35%). Few mothers received education past junior high school (4%), with 40% receiving no formal education. The median daily maximum and minimum WBGT values during the exposure window were 27.2 °C (IQR: 26.7–27.7) and 18.9 °C (IQR: 18.5–19.2), respectively. Figure S4 shows the distribution of daily maximum WBGT for each participant across the full exposure window, as well as stratified by dry and wet seasons. Days exceeding the 99th percentile WBGT were uncommon overall; 39.7% of participants experienced at least one such day, and the median number of days above this threshold was 0 (IQR: 0–4) during the exposure window. Full baseline characteristics of the analytic sample are presented in table 1. Characteristics of GRAPHS participants included versus excluded from these analyses are presented in supplementary table S4. Overall, participants included in the analytic sample were similar to the broader GRAPHS cohort with respect to key demographic and socioeconomic characteristics.

**Table 1.** Participant and exposure characteristics for the analysis sample (*N* = 312). Heat metrics reflect each participant’s mean daily minimum and maximum wet bulb globe temperature (WBGT) averaged from 30 d preconception through 210 gestational days; summary statistics are median (IQR) across participants.

	Median (IQR) or *n* (%)^*a*^
*N*	312
Age (years)	28.0 (23.0, 34.0)
BMI (kg m−^2^)	22.9 (21.2, 24.7)
Number of previous pregnancies	3.0 (1.0, 5.0)

Season of delivery	
Dry (Nov–Mar)	176 (56%)
Wet (Apr–Oct)	136 (44%)

SES (1–5, 1 = least poor and 5 = very poor)	
1	65 (21%)
2	62 (20%)
3	56 (18%)
4	77 (25%)
5	52 (17%)

Marital status	
Married	177 (57%)
Living together	108 (35%)
Single	27 (9%)

Education level	
None	125 (40%)
Primary	89 (29%)
Junior high school	85 (27%)
Secondary high school	13 (4%)
Sum NDS score (continuous)	4.0 (2.0, 5.0)

Sum NDS category	
Low	89 (29%)
Moderate	153 (49%)
High	70 (22%)
Mean daily minimum WBGT (°C)	18.9 (18.5, 19.2)
Mean daily maximum WBGT (°C)	27.2 (26.7, 27.7)

a *Note*. Statistics show median (IQR) or *n* (%).

BMI = body Mass Index; SES = socioeconomic status; NDS = negative domain score; WBGT = wet bulb globe temperature.

The distribution of Sum NDS scores was unimodal and slightly right-skewed, with scores bounded between 0 and 12 (figure S5). The median score was 4 (IQR: 2–5). Based on the predefined thresholds, 89 participants (29%) were classified as low stress (NDS 0–2), 153 (49%) as moderate stress (NDS 3–5), and 70 (22%) as high stress (NDS >5). Figure S6 highlights the domains that primarily contribute to the stress scores, with Panel A showing the number of negatively perceived events in each domain and Panel B showing the number of participants who experienced at least one negatively perceived event for each domain (i.e. what determined the Sum NDS score). Financial events were the most common negatively perceived events, followed by relationship and home. These three domains were the primary contributors to the Sum NDS score as well.

The proportional odds assumption was satisfied for the WBGT term across all five exposure windows (Brant test *p*-values: range 0.12–1.00). Where omnibus tests indicated departures, these were attributable to categorical covariates with sparse cells (education, marital status) rather than to the heat exposure term. Binary recoding at both outcome thresholds yielded WBGT odds ratios consistent with the primary ordinal model, further confirming this assumption (supplementary tables S5–S7 and supplementary figure S7). GVIF values confirmed acceptable collinearity between heat exposure and seasonal adjustment across all specifications (supplementary table S8). Findings were robust to alternative seasonal/calendar-time specifications (supplementary tables S9 and S10 and supplementary figure S8).

Associations between trimester-average daily maximum WBGT and maternal stress

Individual ordinal logistic regressions were run for each pregnancy period, adjusting for covariates. The main results for the daily maximum WBGT averages are presented in table 2, with FDR-adjusted *p*-values reported in supplementary table S11. Results indicate that higher daily maximum WBGT during the first trimester (OR = 1.44; 95% CI 1.15–1.81; *p* = 0.0014) was significantly associated with belonging to a higher stress category. The second trimester association (OR = 1.30; 95% CI 1.01–1.68; raw *p* = 0.038) did not survive FDR correction (p_FDR = 0.063) and should be interpreted with caution. Higher average daily maximum WBGT across the entire observed pregnancy window was also found to be associated with higher stress (OR = 1.64; 95% CI 1.17–2.31; *p* = 0.0040). Preconception and third-trimester average daily maximum WBGT were not found to be significantly associated with stress scores. Table S12 in the supplementary materials contains the full model output with covariates. A model incorporating all pregnancy periods in the same regression (table S13), thereby adjusting each trimester’s estimate for WBGT in the other periods, yielded similar findings. Only first-trimester WBGT was significantly associated with higher stress (OR = 1.47; 95% CI 1.13–1.92; *p* = 0.0043).

**Table 2.** Adjusted associations between average daily maximum WBGT and CRISYS-R stress category across pregnancy exposure windows. Odds ratios represent the change in odds of being in a higher CRISYS-R stress category associated with a one-unit increase in average daily maximum WBGT during each exposure window. Models adjust for maternal age, BMI, socioeconomic status (asset index), number of previous pregnancies, and season of delivery. *N* = 312 pregnancies, except for the third-trimester model, which includes only those surveyed ⩾2 weeks into the trimester (*N* = 276). The ‘entire pregnancy’ window covers 30 d preconception through the survey date. FDR-adjusted *p*-values for the primary family of five exposure windows are reported in supplementary table S11.

Exposure window	OR (95% CI)	*P*-value	*N*
Preconception	1.16 (0.94, 1.43)	0.16	312
First trimester	1.44 (1.15, 1.81)	0.0014	312
Second trimester	1.30 (1.01, 1.68)	0.038	312
Third trimester	0.97 (0.80, 1.18)	0.77	276
Entire pregnancy	1.64 (1.17, 2.31)	0.0040	312

Time-varying associations between daily maximum and minimum WBGT and maternal stress

To assess how WBGT effects varied across gestation, we next fit DLNMs, which estimate changes in the odds of belonging to a higher CRISYS-R stress category across daily maximum and minimum WBGT values and gestational lags, relative to the median WBGT. Slice plots were used to illustrate lag-specific associations at selected WBGT percentiles (1st, 25th, 75th, and 99th percentiles).

**Figure 1.**
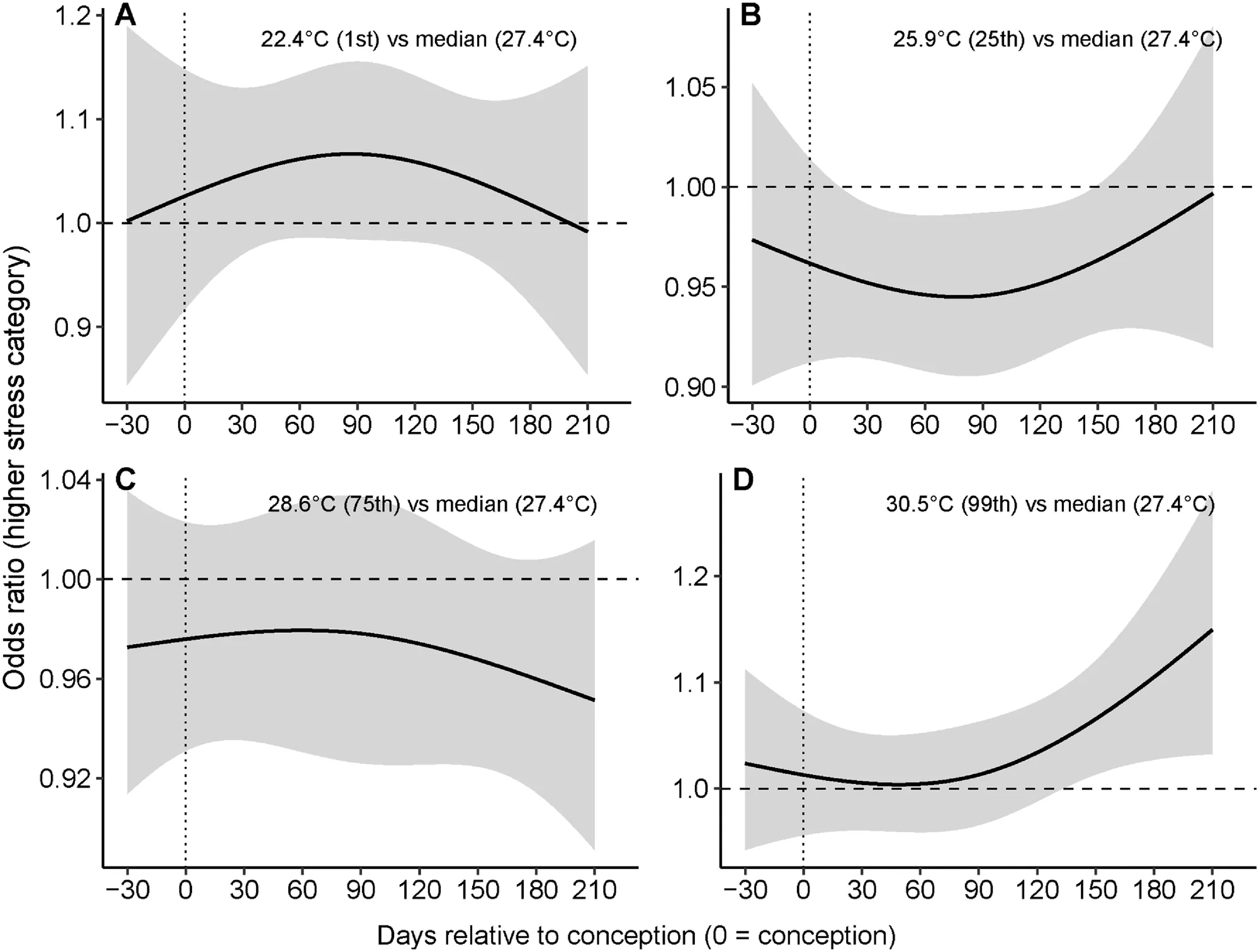
Distributed lag non-linear models demonstrating lag-specific associations between daily maximum WBGT and odds of reporting higher stress defined by the CRISYS-R questionnaire. Curves show the estimated time-varying odds ratio at each day relative to conception (Day 0), comparing four reference temperatures (Panel A: 1st; Panel B: 25th; Panel C: 75th, and Panel D: 99th percentiles of daily maximum WBGT) to the median maximum WBGT (27.4 °C). Shaded regions represent 95% confidence intervals; areas where the confidence intervals do not cross 1 indicate a sensitive window of exposure. Each panel uses an independent *y*-axis scale to preserve detail; a supplementary version with a common axis scale is provided in supplementary figure S21. Pointwise 95% CIs are shown; these do not control the family-wise error rate across gestational lags. Simultaneous Scheffé confidence bands are provided in supplementary figure S23.

For daily maximum WBGT (figure 1), 25th percentile (25.9 °C) values were associated with lower odds of higher stress category from early pregnancy through roughly 150 gestational days. In contrast, 99th percentile (30.5 °C) values were associated with increased odds beginning around 135 gestational days and continuing through later pregnancy. To summarize the magnitude of these associations, we report cumulative effects over 7 d windows at two gestational periods where the 99th-percentile association was evident, which serve as consistent reference points throughout our sensitivity analyses. During gestational days 135–141, exposure at the 99th percentile was associated with 42% higher odds of belonging to a higher stress category compared with median WBGT (OR = 1.42; 95% CI 1.03–1.97); the association strengthened further by days 180–186, reaching roughly twice the odds (OR = 2.07; 95% CI 1.21–3.52). However, under cluster-bootstrap resampling accounting for the limited number of independent exposure series (*N* = 10 grid cells), confidence intervals for these cumulative windows widened to include the null, indicating limited precision; point estimates nonetheless remained stable across resampling and leave-one-out exclusions (supplementary tables S14–S16 and supplementary figure S9). A full exposure–lag contour plot is shown in figure S10.

**Figure 2.**
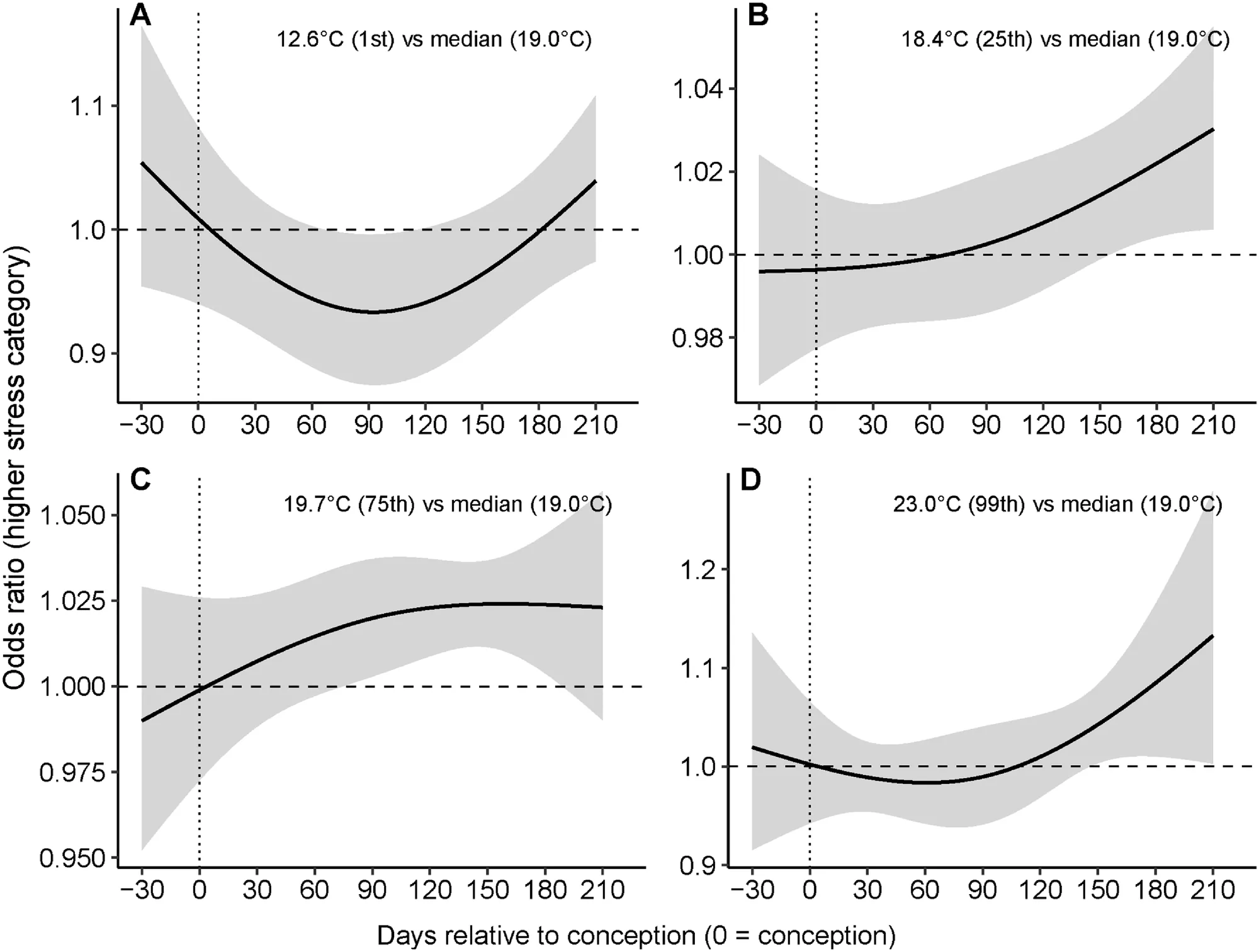
Distributed lag non-linear models demonstrating lag-specific associations between daily minimum WBGT and odds of reporting higher stress defined by the CRISYS-R questionnaire. Curves show the estimated time-varying odds ratio at each day relative to conception (Day 0), comparing four reference temperatures (Panel A: 1st; Panel B: 25th; Panel C: 75th, and Panel D: 99th percentiles of daily minimum WBGT) to the median minimum WBGT (19.0 °C). Shaded regions represent 95% confidence intervals; areas where the confidence intervals do not cross 1 indicate a sensitive window of exposure. Each panel uses an independent *y*-axis scale to preserve detail; a supplementary version with a common axis scale is provided in supplementary figure S22. Pointwise 95% CIs are shown; these do not control the family-wise error rate across gestational lags. Simultaneous Scheffé confidence bands are provided in supplementary figure S24.

For daily minimum WBGT (figure 2), the 1st percentile (12.6 °C) was associated with decreased odds of stress between approximately 65 and 115 gestational days. The 25th percentile (18.4 °C) was associated with increased odds of stress beginning at approximately 155 gestational days. The 75th percentile (19.7 °C) was associated with increased odds of stress from approximately 75–190 gestational days, while the 99th percentile (23.0 °C) was associated with increased odds of stress beginning around 145 gestational days and continuing through the end of the exposure window. The full contour plot is shown in figure S11.

Sensitivity analyses using alternative heat stress metrics showed that associations based on daily maximum HI closely mirrored the primary WBGT results in both timing and direction across pregnancy (supplementary figure S12). Associations based on T2m exhibited overlapping periods of elevated risk but differed in magnitude and percentile-specific patterns, with 99th percentile daily maximum T2m associated with higher stress for most of the exposure window (supplementary figure S13). In contrast, Tw showed weaker and less consistent associations across percentiles, particularly at the upper end of the exposure distribution (supplementary figure S14). For daily minimum temperatures, associations based on HI and T2m closely resembled those observed for minimum WBGT, while minimum T2m exhibited different patterns with greater fluctuations in the estimated odds ratios across gestational lags within each percentile slice (supplementary figures S15–S17).

Time-varying associations between overnight cooling and maternal stress

In a joint DLNM including both daily maximum WBGT and overnight cooling (ΔWBGT), lagged associations at the 99th percentile of maximum WBGT (30.5 °C) were similar to those observed in the primary analysis, with increased odds of stress later in gestation. At the 25th percentile (25.9 °C), the preconception period and early first trimester were associated with decreased odds of higher stress. A similar pattern was observed at the 75th percentile, although the window of significance was shorter, with preconception and initial weeks of first trimester associated with reduced odds of higher stress. Slice plots for this model setup are provided in figure S18.

Impact of WBGT on financial domain stress

Because financial events were the most common contributor to the Sum NDS, we conducted additional analyses focusing on financially related stress. Participants were grouped into low, medium, and high financial stress categories based on the number of negatively perceived financial events reported. The financial stress DLNM pattern mirrored the Sum NDS results at the 99th percentile of daily maximum WBGT: beginning around 125 gestational days, higher daily maximum WBGT was associated with increased odds of belonging to a higher financial stress category. No significant associations were observed at lower daily maximum WBGT percentiles or for other stress domains. Slice plots for the financial analysis are shown in figure S19.

## 4. Discussion

Leveraging a longitudinal pregnancy cohort, we examined associations between heat exposure over pregnancy and maternal stress, identifying that warmer temperatures are associated with higher reported stress while relatively cooler temperatures within the local Ghanaian climate may be protective. Models based on trimester-average daily maximum WBGT suggested that higher temperatures during the first trimester and across the full pregnancy period were associated with higher maternal stress. Using DLNMs, a data-driven approach which allows more flexible analysis of time-varying associations across gestation, we observed that exposure to extreme heat (e.g. 99th percentile daily maximum WBGT) relative to the median was associated with increased odds of higher stress in mid-to-late gestation. Sensitivity analyses using different heat measures produced similar results. These data suggest that public health interventions to reduce psychosocial stress in the perinatal period should consider heat adaptation strategies, especially amongst pregnant populations in lower-resource settings.

Differences between the trimester-average and DLNM results may reflect the seasonal structure of heat exposure, which induces correlation between exposure windows across pregnancy [27]. When trimester-average exposures are correlated, models that examine each period in isolation can misattribute the timing of vulnerability [27]. In addition, trimesters are defined by calendar-based groupings of pregnancy rather than underlying physiological or developmental processes, which may obscure narrower or cross-trimester periods of vulnerability. DLNMs address these challenges by simultaneously accounting for exposure across gestation and allowing associations to vary continuously over time, rather than pre-specifying trimester boundaries [27].

Meanwhile, variation across heat stress metrics likely reflects differences in their construction interacting with the humid tropical context of the study setting. To provide additional context on how these heat stress metrics relate to one another in this setting, figure S20 compares the joint distributions of daily maximum WBGT with HI, T2m, and Tw, stratified by season. Associations based on HI closely mirrored those observed for WBGT, matching expectations as WBGT is derived directly from the HI [21]. Both metrics preserve variability at higher heat levels, resulting in broadly consistent patterns. In contrast, Tw becomes constrained under highly humid conditions, compressing the upper end of the exposure distribution and limiting contrast at higher percentiles, which may contribute to the weaker and less consistent associations observed [16, 28]. Associations based on daily T2m showed partial alignment with WBGT, with both metrics indicating increased stress at the highest exposure percentile. However, associations based on T2m differed from WBGT at the 25th and 75th percentiles, where patterns were less consistent across gestation, suggesting that ambient air temperature alone may capture different aspects of heat exposure than composite metrics that incorporate humidity. Furthermore, it is important to note that while all heat stress metrics are reported in °C, each metric scales differently and thus a 1 °C change in one metric does not correspond to a 1 °C shift in another [16, 28].

While we are not able to attribute specific causal explanations for these results, a number of known biological, psychological, social, and economic pathways exist that support the observed associations [2]. Thermoregulation is affected by temperature and can result in heat exhaustion or heat stroke, which can have long lasting impacts on cerebellar function [2]. Exposure to higher temperatures, particularly when temperatures remain high at night, can result in less sleep and disrupt natural circadian rhythms [29]. Sleep and mental health have been shown to have a reciprocal association, with poor sleep causing mental health issues and vice versa [30]. Psychologically, studies have found conditions such as depression, anxiety, and schizophrenia to increase in heat waves [31].

Social changes during high-heat conditions offer another perspective. Increased temperatures are associated with irritability and even aggression [32]. Additionally, individuals are less likely to spend time outdoors during heat waves, potentially leading to loneliness and isolation [2]. Heat waves have also been shown to influence agricultural output [33]. Financial events were the leading contributor to Sum NDS scores and the GRAPHS cohort is made up of largely agrarian communities, so heat related impacts on crop yield could plausibly amplify heat related stress due to financial impacts, though this pathway was not directly tested in the current analysis.

The mechanisms described above further help contextualize why the regression models identified associations primarily in the first trimester, while the DLNMs highlighted effects of extreme heat later in pregnancy. The regression models reflect average heat exposure over extended pregnancy windows, approximating cumulative heat burden. Since the stress survey was administered months after the first trimester, cumulative heat exposures early in pregnancy could plausibly influence stress measured later in gestation. For example, heat during the first trimester could impact agriculture and income months later [34]. In comparison, the DLNMs capture daily temperatures and are more sensitive to shorter term heat exposures. Overall, these results suggest that longer-term average heat exposure may be more relevant earlier in pregnancy, while extreme heat events may have stronger effects later in gestation.

While our analyses do not examine the downstream maternal-child health impacts of maternal stress, a well-established literature—including studies within the GRAPHS cohort—has linked higher prenatal stress to adverse child outcomes; these include worse birth outcomes such as lower birth weight in addition to later childhood health risks such as reduced lung function and higher asthma risk [18, 35, 36]. Higher stress may also impair maternal health; for example, higher stress has been linked to risk of cardiovascular disease, coronary heart disease, stroke events, diabetes, hypertension, and obesity [37]. A better understanding of the associations between heat and stress during pregnancy may help inform care [13, 36]. Although additional evidence is necessary to inform policy decisions, these findings provide a starting point to guide future research.

Taken together, our results provide new evidence linking heat exposure to maternal stress in tropical low- and middle-income country (LMIC) populations, where such evidence remains limited despite rising temperatures and high vulnerability to heat-related impacts [3, 14]. Understanding the spectrum of health impacts in resource limited settings is critical for directing public health investments. Mental health conditions, including stress, are common in Ghana and across sub-Saharan Africa, with community-based studies documenting substantial prevalence shaped by socioeconomic vulnerability [38, 39]. At the same time, access to mental health care remains scarce in much of the region, and stigma often discourages individuals from seeking support, which constrains care and obscures the true prevalence of mental health conditions [40]. In this context, identifying environmental antecedents of stress, and the gestational periods during which they are most influential, is important for informing interventions that could reduce exposure or buffer stress during pregnancy. By elucidating when heat exposure may influence maternal stress, our findings help identify opportunities for targeted prevention and adaptation strategies in heat-vulnerable settings. Potential intervention strategies in this context include nocturnal cooling measures (improved ventilation, lightweight bedding), housing modifications such as reflective roofing and shaded rest areas, community-level heat alert systems targeting pregnant women through existing antenatal care networks, and agricultural support programs to buffer household income during periods of peak heat exposure in early pregnancy.

Strengths of this study include its focus on a well-characterized pregnancy cohort (GRAPHS) in rural Ghana, an understudied setting with high vulnerability to heat exposure. Maternal stress was assessed using a validated, domain-based instrument, enabling characterization of stress burden and contributing domains. The use of daily WBGT provided a physiologically relevant measure of heat stress, while the application of DLNMs allowed for flexible evaluation of time-varying associations across gestation. These findings may also be relevant to other tropical and subtropical regions experiencing humid heat and similar socioeconomic vulnerabilities.

While this study contributes new evidence on heat exposure and stress during pregnancy in a LMIC setting, there are a number of limitations to consider. The analytic sample size (*N* = 312) is small, limiting precision, particularly at the edges in lag and temperature for the DLNMs. The DLNMs also required identical window lengths, leading us to exclude 41 women from the analytic sample and to restrict the lag window to two weeks into the third trimester. This prevented us from identifying associations closer to the delivery date. Future studies could strengthen this analysis by utilizing a larger sample size and surveying participants closer to the delivery date or in the postpartum period.

A key spatial limitation of this study is the coarse resolution of ERA5 reanalysis data (0.25° × 0.25°, ∼25 km), which resulted in 41 communities mapping to only 10 distinct grid cells. Participants sharing a grid cell had correlated daily exposure histories, reducing the effective number of independent exposure series. Primary analyses use model-based standard errors at the individual level, which is standard practice when ERA5 is used as an ecologically-assigned exposure predictor. A cluster bootstrap sensitivity analysis confirmed that point estimates were robust to this exposure structure, though confidence intervals widened substantially with only 10 independent clusters (supplementary tables S14–S16 and supplementary figure S9). We therefore interpret our precision estimates with appropriate caution and note that higher-resolution meteorological data, including hourly readings and a full suite of meteorological variables, are lacking for Ghana, representing an inherent constraint of environmental epidemiology in this setting. The shared exposure structure within grid cells may partially strengthen causal inference by ensuring that within-cell variation in stress outcomes cannot be attributed to differences in ambient climate conditions; the associations identified therefore reflect temporal variation in WBGT across gestation rather than spatial differences between communities.

Beyond spatial resolution, our exposure metric has inherent constraints. Although shaded WBGT approximates heat exposure in shade rather than under direct sun [22], the underlying ERA5 data provide an outdoor ambient exposure measure; actual personal exposure will vary by time spent outdoors, housing quality, and behavioral adaptation strategies such as shade use or ventilation, none of which were measured in this cohort. While shaded WBGT [22] is an established WBGT metric used in environmental epidemiology research [41], it does not account for solar radiation and assumes a fixed wind speed. Because it excludes direct solar load, it is a conservative measure that likely underestimates true outdoor heat exposure, particularly for women spending substantial time in agricultural work; exposure error of this kind would generally tend to attenuate rather than exaggerate the observed association.

A further limitation concerns the stress instrument. Since the CRISYS-R records life events occurring within the prior six months without dating individual events, our outcome reflects a single assessment integrated over a broad window; lag-specific associations should therefore be interpreted as reflecting broader exposure periods rather than precise temporal timing, and sensitivity analyses restricted to shorter recall windows are not possible. We cannot therefore formally exclude the possibility that the lag-response pattern in figure 1(D) is partly attributable to telescoping bias, whereby events proximal to the survey date are more readily recalled. However, the absence of a parallel monotonic increase at other temperature percentiles, and the stronger association observed in the first trimester than the third in trimester-average models, argue against recall bias as the primary explanation. Prospective cohort designs with event-timed stress measurement will be needed to formally disentangle gestational timing effects from recall processes in this and similar populations. A follow-up study currently in development by our group and collaborators (the West Africa Centre for Climate and Health Innovation and Resilience) will prospectively examine heat exposure, maternal mental health, and the mechanisms linking them; such prospective designs may help address the temporal-resolution limitations of a single retrospective stress assessment.

Finally, although sensitivity analyses across alternative seasonal adjustment specifications produced broadly consistent findings, and collinearity between heat exposure and seasonal terms was negligible (GVIF range: 1.05–1.20), residual confounding by season-dependent factors, such as agricultural cycles and associated financial stress, cannot be fully excluded with observational data.

## 5. Conclusion

In this analysis, both trimester-averaged temperatures and daily heat exposures showed significant associations with maternal stress, suggesting that sustained heat early in pregnancy and extreme heat later in gestation may be particularly impactful. As temperatures continue to rise across sub-Saharan Africa, a region critically underrepresented in existing research on climate and maternal health, identifying when pregnant individuals are most vulnerable to heat exposure is important for understanding and addressing maternal heat burden. While additional research is needed to clarify underlying mechanisms and to evaluate potential interventions, this study provides new evidence on the timing and magnitude of heat-related stress during pregnancy and offers a foundation for future research and adaptation strategies in heat-vulnerable settings.

## Data Availability

The data cannot be made publicly available upon publication because they contain sensitive personal information. The data that support the findings of this study are available upon reasonable request from the authors, subject to institutional approvals and a data access agreement; requests should be directed to Kenneth A. Ae-Ngibise (kenneth.ae-ngibise@kintampo-hrc.org). Statistical analysis code is available via GitHub. Supplementary Appendix available at: https://doi.org/10.1088/2752-5309/ae90dc/data1.
